# Data driven identification of international cutting edge science and technologies using SpaCy

**DOI:** 10.1371/journal.pone.0275872

**Published:** 2022-10-12

**Authors:** Chunqi Hu, Huaping Gong, Yiqing He

**Affiliations:** 1 School of Public Policy and Administration, Nanchang University, Nanchang, Jiangxi, China; 2 School of Economics and Management, Nanchang University, Nanchang, Jiangxi, China; University of Salento, ITALY

## Abstract

Difficulties in collecting, processing, and identifying massive data have slowed research on cutting-edge science and technology hotspots. Promoting these technologies will not be successful without an effective data-driven method to identify cutting-edge technologies. This paper proposes a data-driven model for identifying global cutting-edge science technologies based on SpaCy. In this model, we collected data released by 17 well-known American technology media websites from July 2019 to July 2020 using web crawling with Python. We combine graph-based neural network learning with active learning as the research method in this paper. Next, we introduced a ten-fold cross-check to train the model through machine learning with repeated experiments. The experimental results show that this model performed very well in entity recognition tasks with an F value of 98.11%. The model provides an information source for cutting-edge technology identification. It can promote innovations in cutting-edge technologies through its effective identification and tracking and explore more efficient scientific and technological research work modes.

## 1 Introduction

Science and technology often characterize a country’s soft power. How practitioners can provide better and more efficient services to promote science and technology has become a critical topic of research and discussion [1]. There is no doubt that there is a maximum effect on scientific research and development today. Whether from the perspective of the investment scale of scientific research and development or the final achievements of scientific research and development, the scientific research development strategies of Europe, America, Japan, and some emerging economies should be focused on. Therefore, analyzing the authoritative texts on R&D strategies or plans of major countries or regions is essential. Secondly, as capital is very sensitive to frontier technology research and development, many influential mainstream institutions have been paying attention to the innovation of emerging technologies and their business models for a long time. They have a good insight into the industrialization development prospect of technology. Therefore, analyzing the relevant reports on leading-edge technology by mainstream consulting agencies is equally important. This study analyzes and discusses the efficient use of scientific and technological information.

Science and technology are how humans perceive the world, and academic literature is the primary way for scholars to exchange ideas. Bibliometrics shows that scholarly literature is growing at a rapid rate of 8%-9% per year, presenting a booming scene of scholarly communication. However, extracting adequate information from the information overload literature has become a central issue in academic research. In recent years, literature analysis through computer technology has received widespread attention. Literature [2–4] summarized the research methods through a comprehensive literature review and identified the latest trends in their respective research fields. The literature [5, 6] revealed the citation link strength (CLS), co-citation relationships of the literature, and its research frontiers with the help of CiteSpace. The literature [7] used patent analysis and other methods to construct a visualized high-tech prospective risk study. The literature [8] extracted metadata from the literature for studying the imagined hyperspectral remote sensing status and its research frontiers. The literature [9] introduced a method incorporating improved technical entropy analysis to identify, measure, and explain topics’ evolution in the graphene literature. By constructing a corpus, the literature [10] used Splunk to detect science and technology fronts and hotspots. The literature [11] discovered the social sentiment of hot topics in science and technology by constructing an undirected weighted network. The literature [12] reveals the reliability of coarse-grained research methods in social media by collecting and analyzing large amounts of content. Literature [13–19] introduced the lack of scalability and efficiency of retrospective content analysis methods only coarse-grained. In contrast, fine-grained analysis is more effective in solving practical problems in opinion monitoring and sentiment analysis.

Technical monitoring of text resources for specific fields is standard in scientific and technical intelligence work. How to efficiently conduct hotspot research for text resources in different segments is one of the main problems that need to be solved by current scientific and technical workers. The aspects of the existing research that need improvement are:

1. Science and technology practitioners commonly use academic journals as the primary data source. This causes a lack of multi-source data analyses that make the findings insufficient or inaccurate. There is also a time lag when using only journal articles in topic cluster analysis.
2. Many scholars try to identify research hotspots by the co-citation relationship of the literature. Still, this approach cannot exclude whether there is personal friendship among co-cited authors for citation, and thus may lead to inaccuracy of the analysis.
3. Unlike the traditional coarse-grained classification, the number of subdivision categories makes it difficult to effectively outline specific category details without complete research, even with the participation of domain experts.
4. Although practitioners can use new technologies, such as big data techniques, to identify and summarize scientific and technical information on the Internet, complete and high-quality annotated data remains the most significant barrier to the widespread use of many deep learning methods.

## 2 Related work

In the era of the new scientific and technological revolution, the scientific and technological community, the business community, the government, and the whole society are increasingly concerned about the development of the frontier of science and technology, expecting to make a reasonable layout and seize the opportunity through the prediction of future science and technology. For the prediction of future technology, a relatively systematic method has been formed, including qualitative prediction, quantitative prediction, timing prediction, probability prediction, and so on. Currently, most research on specific domains is dominated by supervised learning methods. Although it achieves better results at this stage, it relies on a large amount of manually annotated corpus, making it challenging to achieve efficient and high-quality training on large amounts of data.

Considering that the focus of this research is based on reality to excavate the cutting-edge hot spots of science and technology, that is, the focused problems in various fields of science and technology, key research technologies. This section will discuss the methods that can solve the above problems.

### 2.1 Data-driven

Multi-source fusion of data of different dimensions has become a distinctive feature in the era of big data [20]. A data-driven method can collect and process scientific and technological information resources through correlation analysis of all relevant data [21]. Full-sample data analysis provides more comprehensive and objective data support. Through the integration of deep learning and other methods, the method significantly improves the quality and analysis efficiency of information [22].

Modern think tanks need to adjust to data-driven thinking [23]. With the help of big data and optimization technologies, we can conduct accurate analyses and knowledge minings of massive and multi-structured data.

### 2.2 Natural language processing

Natural language processing (NLP) is a branch of artificial intelligence (AI) and machine learning (ML) that can help computers understand, interpret, and manipulate human language [24]. One of the tasks in natural language processing is to find named entities in the text and classify them into specific predefined categories [25]. Some applications and usages of machine learning, such as NLP, are based on supervised learning [26].

### 2.3 Semi-supervised learning methods

Since unsupervised learning methods [27, 28] may overlook helpful information due to too few samples of category labels, thus leading to inaccurate results. In this context, domestic and foreign scholars have proposed semi-supervised learning methods. Semi-supervised learning is a branch of machine learning that involves using labeled and unlabeled data to perform specific learning tasks. Conceptually, it lies between supervised and unsupervised learning, allowing the use of large amounts of unlabeled data available in many use cases and typically smaller labeled data sets. The literature [29] proposed a semi-supervised learning framework (Unsupervised Data Augmentation, UDA) with good results and a simple framework. Experimental results proved to exceed the effectiveness of fully supervised learning. The literature [30] proposed a semi-supervised SVM-based feature selection (S3VM-FS) model based on a support vector machine (SVM) for gene expression data analysis. Since high-quality labeled documents are complicated to obtain in the NLP domain, researchers have started to apply semi-supervised learning techniques to the NLP domain.

### 2.4 Self-supervised learning methods

Self-supervised learning can capture and use the dependencies between different dimensions of training data by defining training tasks. It focuses on solving the problem of how to improve the effectiveness of machine learning models in the presence of high labeling costs. Current research has demonstrated that self-supervised learning has achieved impressive performance on different downstream tasks, such as language sequences and target recognition.

### 2.5 Label propagation algorithm

The label propagation algorithm [31] is a classical graph semi-supervised method whose core idea is to class propagate unlabeled samples by constructing a similarity matrix among all samples with the labels of the labeled samples as constraints. There are many methods to construct similarity matrices, such as k-nearest neighbor methods [32], local linear representation methods [33], low-rank representation methods [34], heat kernel methods [35], and sparse representation methods [36].

### 2.6 Active learning method

Active learning is a human-computer interactive iterative training method. It considers how to select labeled samples among unlabeled samples with labeling values [37], hand over these samples to corresponding experts for labeling, and then participate in model training, which is used to improve the learning efficiency of the model and reduce the waste of labeling resources. The idea of combining deep learning and active learning was probably first proposed in 2014 by kind of literature [38, 39] proposed a recent Bayesian generative active deep learning model. The literature [40] proposed a two-step iterative approach for mobile and active learning in remote sensing. The literature [41] proposed an approach by incorporating the intrinsic distribution information of unlabeled samples into the metric parameters of the samples.

### 2.7 SpaCy

In 2016, Explosion launched SpaCy [42]. SpaCy is an open-source natural language processing library that supports a variety of tasks, including part-of-speech tagging, dependency analysis, named entity recognition, etc. Its performance in these aspects [43] is excellent. SpaCy can use complex neural network-based models to implement natural language processing components. These components achieve the most advanced results for many tasks and have the advantage of integrating word vectors. This new tokenization algorithm improves performance and ease of definition by aligning the original string to achieve a better balance. It is currently the world’s fastest and can be used for Python’s and Cython’s advanced natural language processing libraries. It is also the best method for deep learning of text. It can seamlessly integrate with other outstanding AI ecosystems such as TensorFlow, PyTorch, Scikit-learn, Gensim, and Python. In the newly released SpaCy, the deep learning model is ten times smaller than the previous generation model, the accuracy rate is 20% higher, and the running cost is lower [44]. According to research, the new SpaCy is fast and performs well when using similar tools and supporting similar functions.

## 3 Materials and methods

Currently, two main research methods to obtain frontier areas from the scientific and technical text information.

Firstly, natural language processing is based on scientific and technical text data, which can be written as *f*(*x*)→*M* only the semantic information *x* of scientific and technical text is utilized. The text feature representation matrix M is output by the neural network *f*. Any row *M*_*e*_ in the matrix *M* can be used to represent a feature representation vector of the corresponding text *e*. This vector can be applied in downstream tasks such as text classification, retrieval, and recommendation.

Secondly, the learning of technological text representation based on graph data, which can be written as *f*(*x*, *I*)→*M*, can utilize not only the semantic information of text *x*, but also the structural information of text network *I*. The core idea is that the characteristics of scientific and technical texts can be expressed to a certain extent by the structure of relationships between scientific and technical texts. The core idea is that technological text features can be expressed by the structure of relationships between technological texts. It mainly uses graph neural network models to extract adequate information from the relational network of scientific and technical texts. It then encodes scientific and technical texts into low-dimensional vectors. Graph neural networks are classified into: supervised, semi-supervised and unsupervised. Since the representation based on semantic information of text has inherent disadvantages, this paper focuses on the research method based on neural graph networks combined with active learning.

SpaCy, as a natural language learning approach, has components that can be individually updated to suit specific task implementations. The NER component of the SpaCy pipeline is a deep learning model using convolutional neural networks and long and short-term memory architectures. It has been shown that the literature [45] used SpaCy to obtain semantic feature vectors of documented texts. The literature [46] designed and implemented a noun term entity recognition method using SpaCy. The literature [47] used the spaCy pipeline to propose that a healthcare model trained using only 50% of the available training data outperformed 100% of the trainable dataset. The literature [48] compares the effectiveness of using NLTK, Stanford CoreNLP, and SpaCy approaches in monitoring data privacy issues. The literature [49] shows that the accuracy of using SpaCy in socially aware training models essentially beats the use of other methods. The literature [50] designed models based on SpaCy with higher accuracy while guaranteeing the same recall rate. Therefore, this article uses SpaCy libraries to train the model.

This paper proposes the following solutions for the hot research in a specific technology domain. First, select the Internet technology news text as the data source(URL: osf.io/vf52s/). The analysis results after three iterations by domain experts are used as the basis for normalized labeling of the newly crawled data, which consists of English texts from science and technology media websites. Second, a focused entity identification algorithm for authoritative technology websites is proposed to pre-filter news sentences that do not contain essential entities to improve the model’s efficiency and accuracy, supplemented by manual review. The model results are then supplemented with expert confirmation to form the final technology hotspots of the year. Finally, international Internet science and technology identification platform for automatic acquisition and analysis of science and technology intelligence information was designed and implemented. The technical architecture, data structure, and internal and external interface design of the platform were completed.

### 3.1 Experimental design and implementation

1. Data pre-processing. Based on the graph neural network algorithm, pre-processing operations such as de-weighting and cleaning are performed on the obtained pre-feed data of 17 Internet science and technology media.
2. The SpaCy algorithm is used to represent news texts. Document vectors are added for each news document to achieve vector conversion of sentences, paragraphs, and whole articles for responding to semantic information.
3. Topic clustering is performed on the obtained Internet technology news texts, and the clustering classes are determined by comparing them with the classification results after expert iteration.
4. The initial classification obtained by training is used to determine the categories of a large number of unlabeled news, and active learning is carried out in a targeted manner for classes prone to misclassification to filter out high-quality samples. Finally, a better model is trained to yield satisfactory experimental results with the computational performance.

### 3.2 Experimental environment

The computer configuration used in this experiment is introduced as follows:

CPU: 2.6GHz six-core Intel Core i7;

GPU: AMD Radeon Pro 5300M 4GB;

Memory: 16GB 2666MHz DDR4;

Operating system: MacOS 10.15.7.

Deep learning training environment: SpaCy version 2.3.1.

### 3.3 Model training

**Fig 1:**
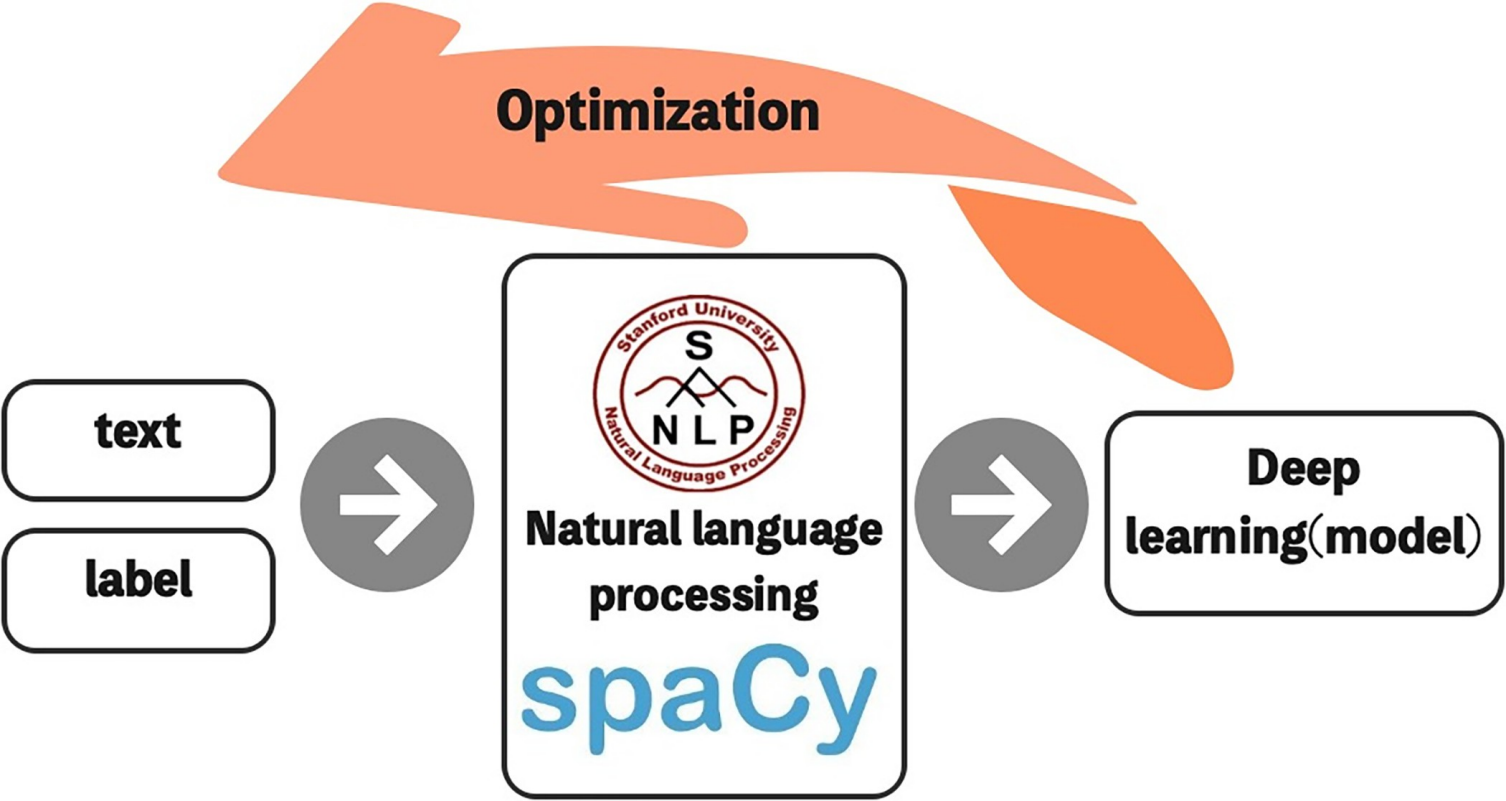
Training process of the international cutting-edge technology recognition model.

SpaCy’s models are statistical. Every "decision" they make (for example, whether a part-of-speech tag or word is a named entity) is a prediction based on all examples used by the model during the training process. In this study, the authors labeled data entities from 17 websites, stored them in the corpus, and used Plac for training. The model training process is shown in **Fig 1**.

In order to make the SpaCy model more representative, the author compared the differences between the models that use single website data to train alone and multi-site data unified training. Many exploratory experiments revealed that the more examples, the more representative the model, and the better the prediction effect.

This study uses the Leader Boards of Technology Media Leaders released by Techmeme, well-known technology news and blog aggregation site in the United States, as a reference [10] (shown in **Table 1**). A total of 17 internet technology media that provide English texts were selected, and Python was used to collect corpus data from the websites and crawl content, themes, titles, and abstracts of all text information from July 2019 to October 2020.

**Table 1:** Ranking of technology websites.

Data source	Website address	Techmeme ranking and well-known technology websites
Arstechnica	https://arstechnica.com/science/	17
Theverge	https://www.theverge.com/	1
Engadget	https://www.engadget.com/	28
Arstechnica_tech	https://arstechnica.com/gadgets/	17
Techcrunch	https://techcrunch.com/	4
Cnet	https://www.cnet.com/	25
Vice	https://www.vice.com/	13
Geekwire	https://www.geekwire.com/	44
Venturebeat	https://venturebeat.com/	19
Fortune	https://fortune.com/	46
Theinformation	https://www.theinformation.com/	15
Fastcompany	https://www.fastcompany.com/	42
Zdnet	https://www.zdnet.com/	12
Reuters	https://www.reuters.com/	6
Gizmodo	https://gizmodo.com/	Well-known technology blogs in the United States
Scientificamerican	https://blogs.scientificamerican.com/	Popular high-level academic journals
Entrepreneur	https://www.entrepreneur.com/	News site about entrepreneurs, small business management and business opportunities
Readwirte	https://readwrite.com/	Internet famous technology news blog

In this study, we wrote a Python program to crawl the relevant content of 17 Internet technology websites. The crawling method used the Arstechnica website as an example.

### 3.4 Named entity annotation

As the basis of machine learning, the automated online processing of text information requires named entity annotation to understand text resources [51]. At the same time, the quality of named entity annotation also affects how efficiently and thoroughly information is obtained. The development of named entity recognition has roughly gone through three stages: from the early lexicon and rule-based approaches to statistical machine learning approaches to deep learning-based approaches in recent years. Similar to Cao Lei [10], this paper will use three iterations in the experiments. The results are used as the standardized labeling basis for the newly crawled data. The newly crawled data consists of the English text from the technology media website. According to the Internet Data Center (IDC) report, it is estimated that by the end of 2025, only 15% of the data can be labeled and analyzed [52]. SpaCy has a high-speed and efficient statistical entity recognition system that assigns tags to a continuous token range. At the same time, SpaCy has the advantage of being able to add any class and new example sentences to the entity recognition system to update the model. Therefore, to reduce the time cost of manually labeling large amounts of data, this paper uses SpaCy and machine learning to automatically label 1565 scientific and technological example sentences on the 17 websites. At the same time, supplemented by manual inspection and review, SpaCy verifies the labeling of named entities. We take The Verge Science as an example below.

    (

    "The method is similar to the voice detection software used by digital assistants like Alexa and Siri, explains Perol",

    {"entities": [(62, 80, LABEL)]}),

  (

    "This isn’t the first time DeepMind’s AI expertise has been used in this way",

    {"entities": [(37, 39, LABEL)]}),

  (

    And Google applied for a patent for a system that uses artificial intelligence trained on electronic health records to build models that could warn doctors of dangerous medical events",

    {"entities": [(55, 78, LABEL)]}),

  (

    "AI algorithms need to be trained in culturally specific ways",

    {"entities": [(0, 13, LABEL)]}),

    (

    "Drone fitted with terrifying claw snatches objects at high speed",

    "entities": [(0, 5, LABEL)]}),

### 3.5 Code implementation

**Obtain the list of information to be crawled for international cutting-edge technology **.

The code is based on the Arstechnica website.

def get_page(page):

url = "https://arstechnica.com/science/page/{}/".format(page)

**Grab the link, Id, picture, date, number of comments, abstract, title, author, picture code**.

results = soup_page.find("main").find_all("li",attrs = {"class":"tease"})

    for result in results:

        #print(result)

        img = result.find("figure").div.attrs["style"].split("\‴)[1]

        link = result.find("figure").a.attrs["href"]

        title = result.find("header").h2.a.string

        abstract = result.find("header").p.string

        if None = = result.find("header").find("p",attrs = {"class":"byline"}):

            continue

    author = result.find("header").find("p",attrs = {"class":"byline"}).find("span").string

    date = result.find("header").find("p",attrs = {"class":"byline"}).find("time").string

comment_count = result.find("footer").find("span",attrs = {"class":"comment-count-number"}).string

    id = hashlib.md5(link.encode("utf-8")).hexdigest()

    data = {}

    data["link"] = link

    data["id"] = id

    data["date"] = date

    data["comment_count"] = comment_count

    data["abstract"] = abstract

    data["title"] = title

    data["author"] = author

    data["img"] = img

**The detailed information page captures the subject, date, body content, and source code of the article**.

paragraphs = article.find("div",attrs = {"itemprop":"articleBody"}).find_all("p")

    content = ""

    for paragraph in paragraphs:

        children = paragraph.children

        for child in children:

            if None! = child.string:

            content = content + child.string

    target["text"] = content

    target["describe"] = describe

    success + = 1

    result.append(target)

    crawlered.append(id)

    dump_count + = 1

    if dump_count = = dump_duration:

        print("Saving data to json file ")

        dump_json()

        print("Saved")

        dump_count = 0

    return

### 3.6 Experimental results

This article uses three indicators, P, R, and F, to measure the performance of entity recognition.


$$P=\frac{n_{i}}{n_{c}},$$ (1)

$$R=\frac{n_{i}}{n_{j}},$$ (2)

$$F=2\times \frac{P\times R}{P+R},$$ (3)

The formula, ***n***_***i***_ represents the number of correctly identified named entities, ***n***_***c***_ represents the number of extracted named entities, and ***n***_***j***_ represents the number of named entities in the corpus.

The SpaCy model was tested using a 10-fold cross-validation process. The specific performance is shown in **Table 2**. Among the ten groups of data, the 10th group has the best experimental evaluation results, with an optimal F value of 98.11%. The precision rate (P), recall rate (R), and F value are 90.63%, 90.74%, and 89.31%, respectively, indicating that the model has achieved good prediction results. In the SpaCy model, the gap between the F value of the 10th group (highest) and the F value of the 3rd group (lowest) is up to 13.84%, which suggests that the quality of the corpus dramatically impacts the performance of SpaCy.

**Table 2:** Automatic extraction and evaluation of international cutting-edge technology recognition entities based on the SpaCy model.

Number	P(%)	R(%)	F(%)
**1**	89.14	86.27	85.71
**2**	89.00	93.85	91.37
**3**	84.07	84.49	84.27
**4**	92.08	90.87	91.47
**5**	91.35	87.5	89.39
**6**	84.72	86.67	85.69
**7**	93.30	91.50	92.39
**8**	92.72	97.02	83.21
**9**	91.93	91.01	91.46
**10**	98.01	98.21	98.11
**Average**	90.63	90.74	89.31

**Fig 2:**
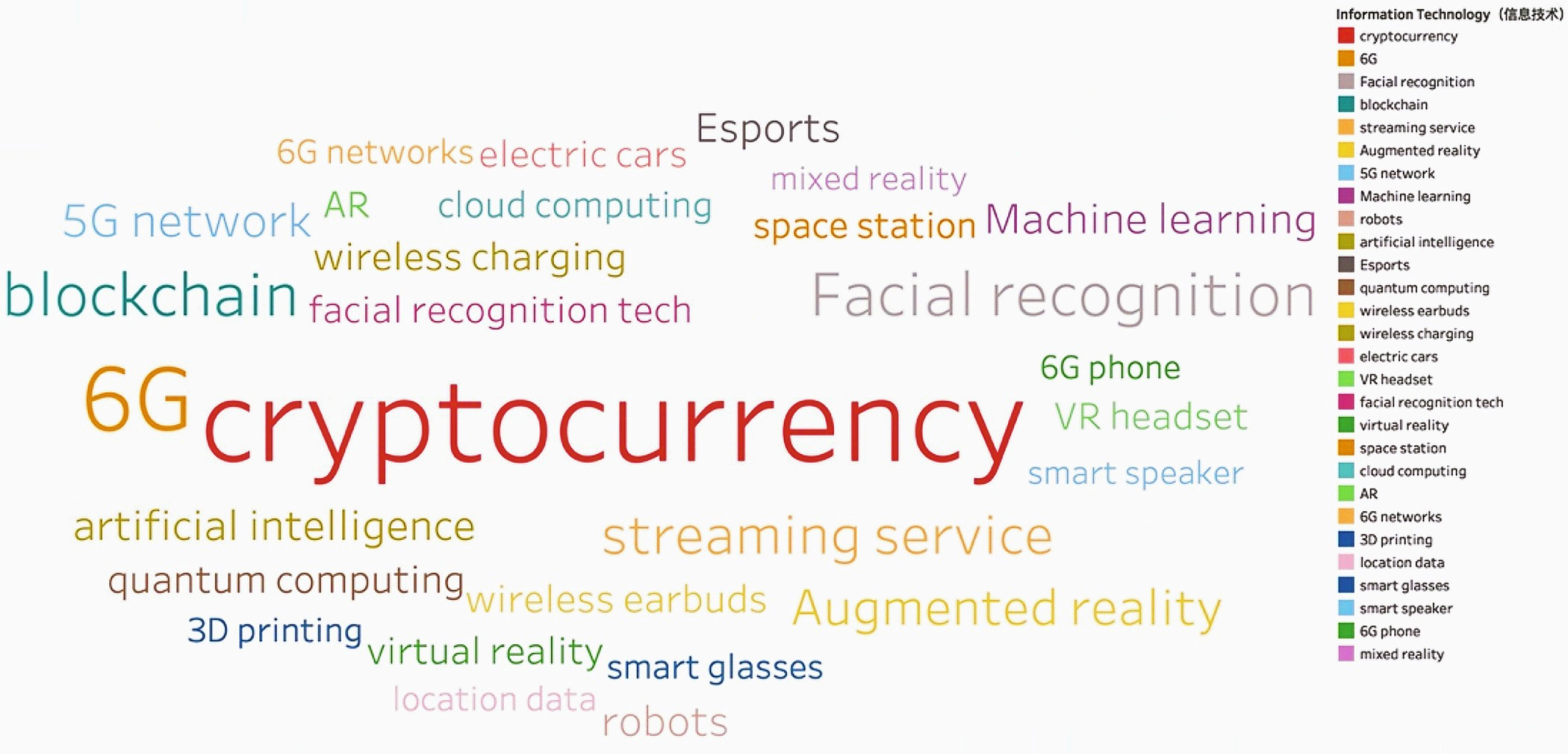
Word cloud diagram in the field of Information Technology.

**Fig 3:**
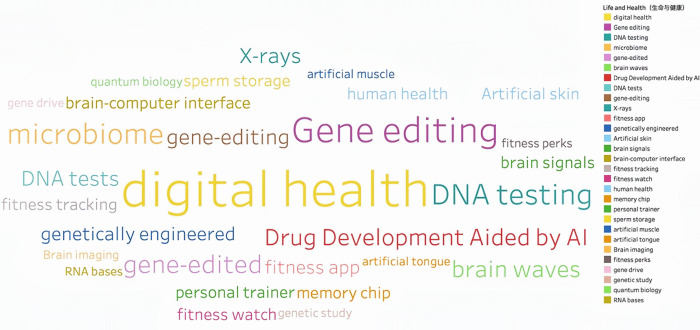
Word cloud diagram in the field of Life and Health.

**Fig 4:**
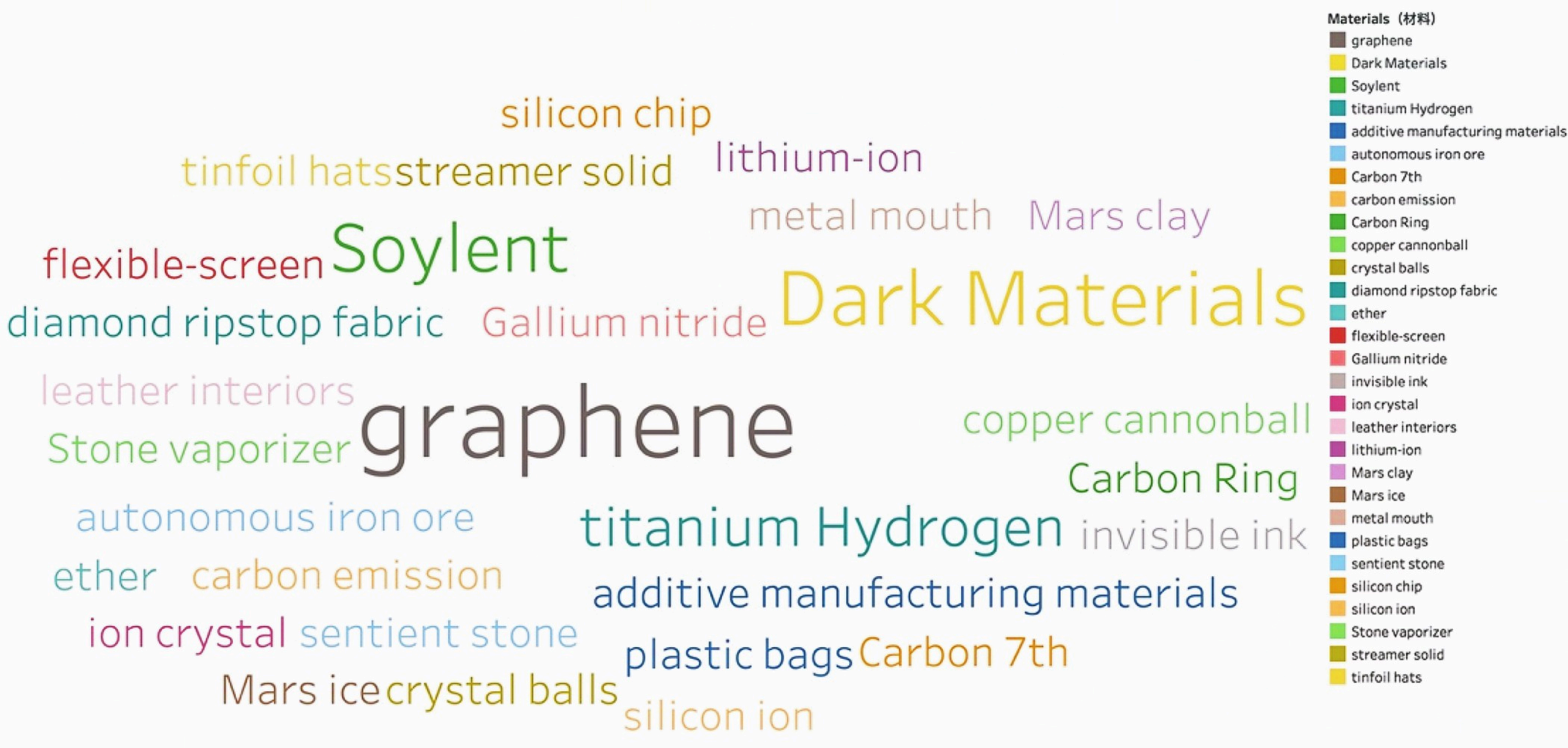
Word cloud diagram in the field of Materials.

**Fig 5:**
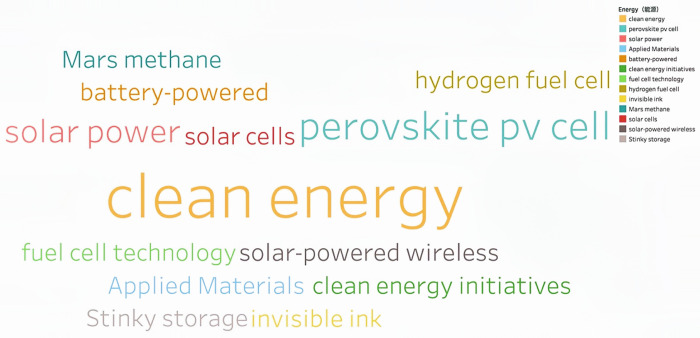
Word cloud diagram of Energy field.

**Fig 6:**
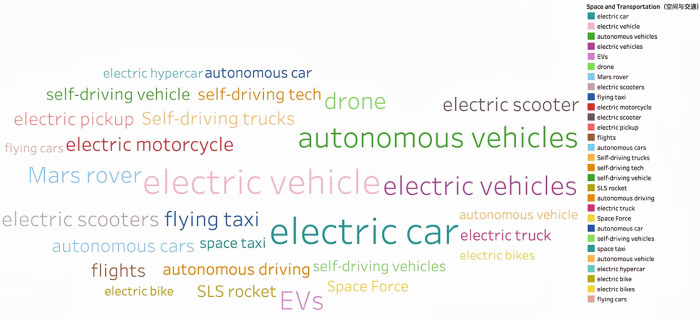
Word cloud diagram in the field of Space and Transportation.

**Fig 7:**
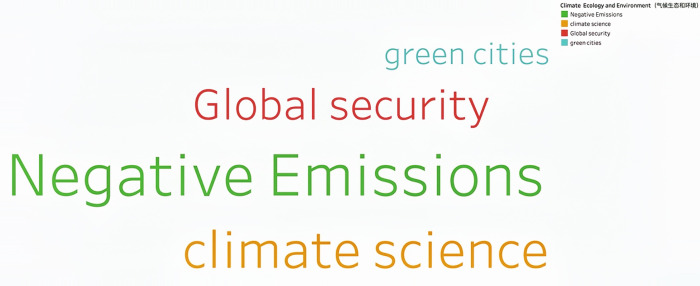
Word cloud diagram in the field of Climate Ecology and Environment.

**Fig 8:**
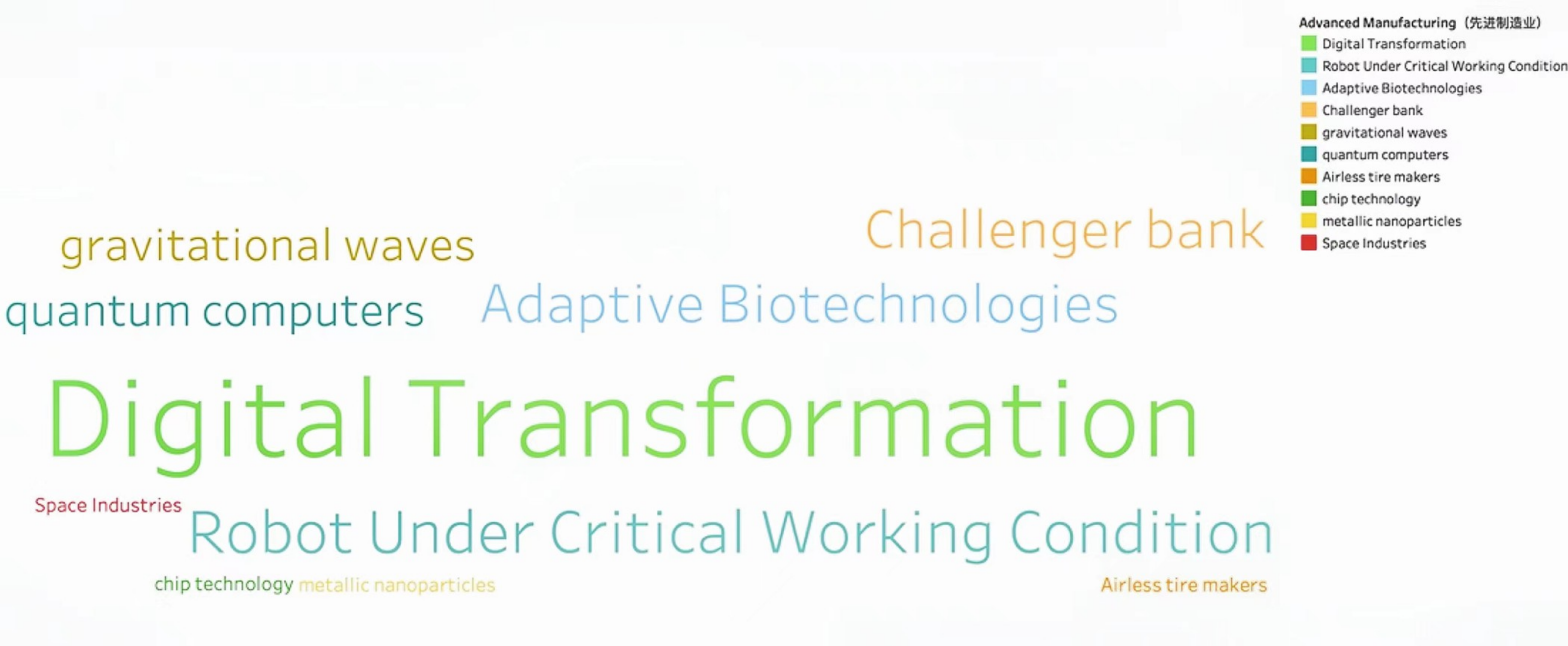
Word cloud diagram in the field of Advanced Manufacturing.

Through continuous training of the model 146,929 keywords were generated. Based on the work experience and classification requirements of the Shanghai Institute of Science and Technology Information, they are now divided into Information Technology, Life and Health, Materials, Energy, Space and Transportation, Climate Ecology and Environment and Advanced Manufacturing in seven fields. **Figs 2–8** show the word cloud diagrams of keywords in various fields sorted by frequency.

### 3.7 Platform construction

**Fig 9:**
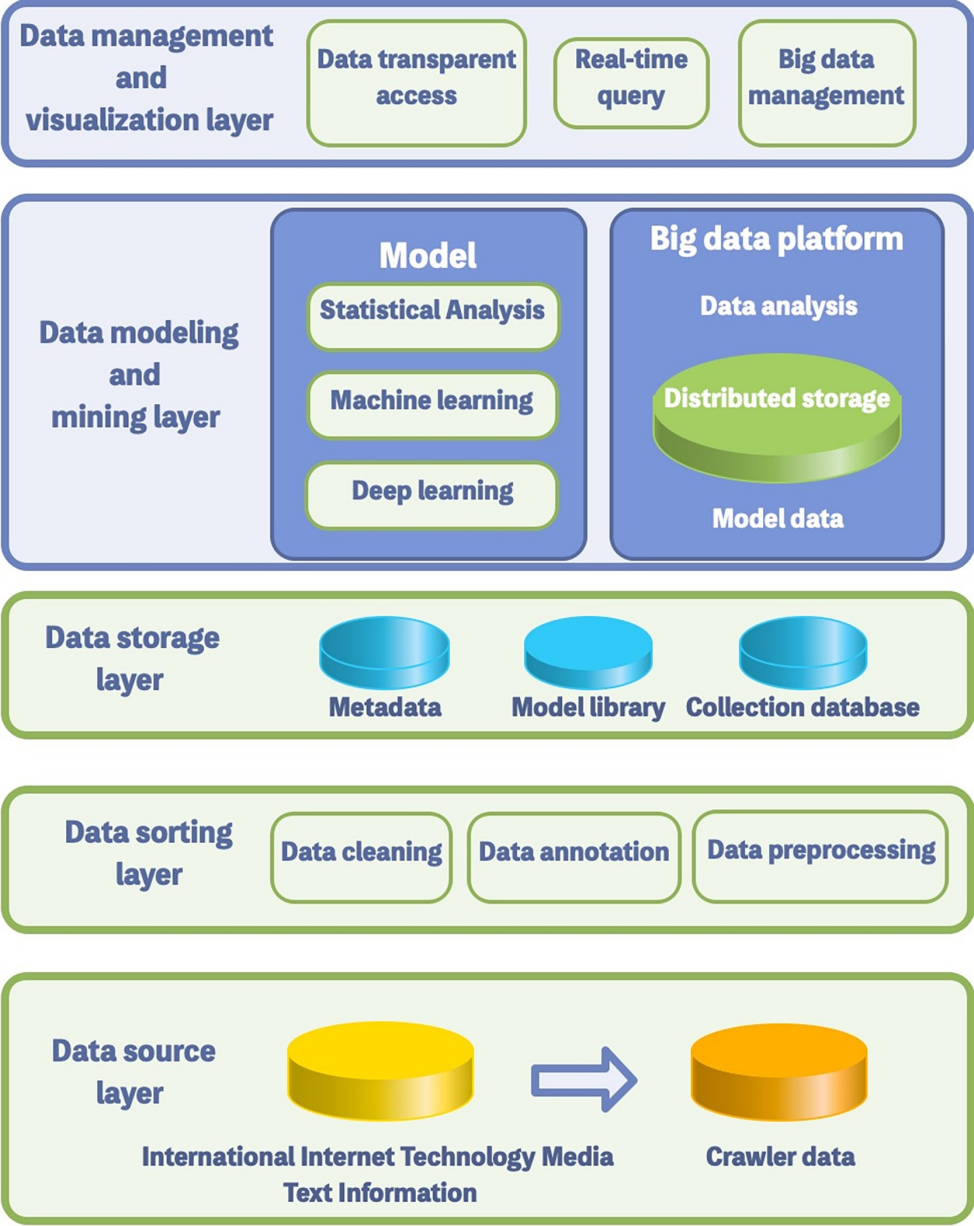
Platform architecture for technology identification.

Based on the information needs, the platform architecture for constructing international cutting-edge technology identification is shown in **Fig 9**. From bottom to top, they are data source layer, data sorting layer, data storage layer, data modeling and mining layer, and data management and visualization layer.

Firstly, data source layer: Based on information needs, we obtain data from 17 international Internet technology media.

Secondlly, data sorting layer: We clean the crawled data and perform named entity labeling and preprocessing.

Thirdly, data storage layer: After cleaning, this layer stores standardized data that can be used for data analysis, such as metadata, model database, collection database.

Fourth, data modeling and mining layer: This layer carries out deep processing of data, establishes statistical analysis models and big data platforms, and uses data mining, deep learning and other algorithms to mine the inherent information.

**Fig 10:**
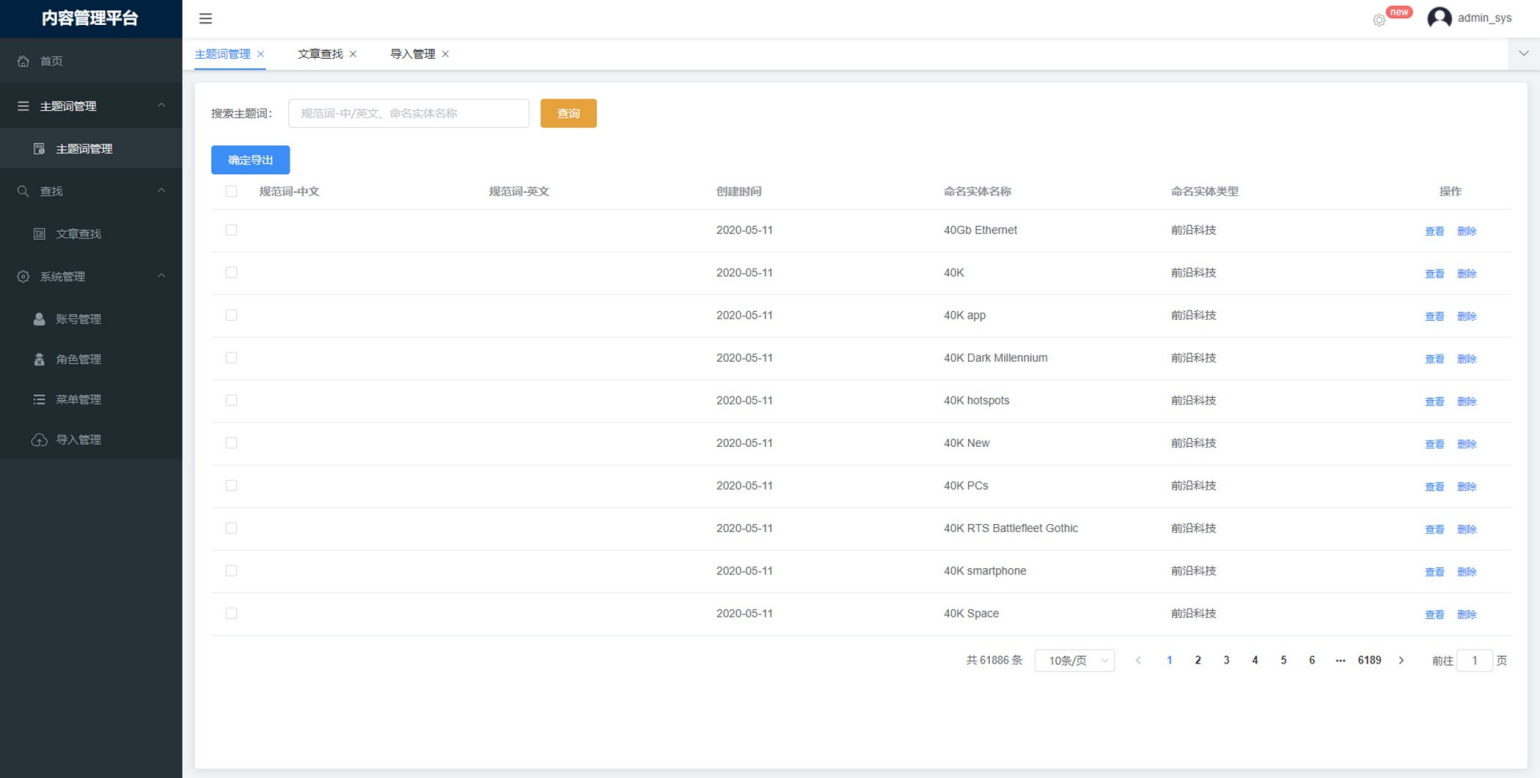
International cutting-edge technology identification subject word management.

**Fig 11:**
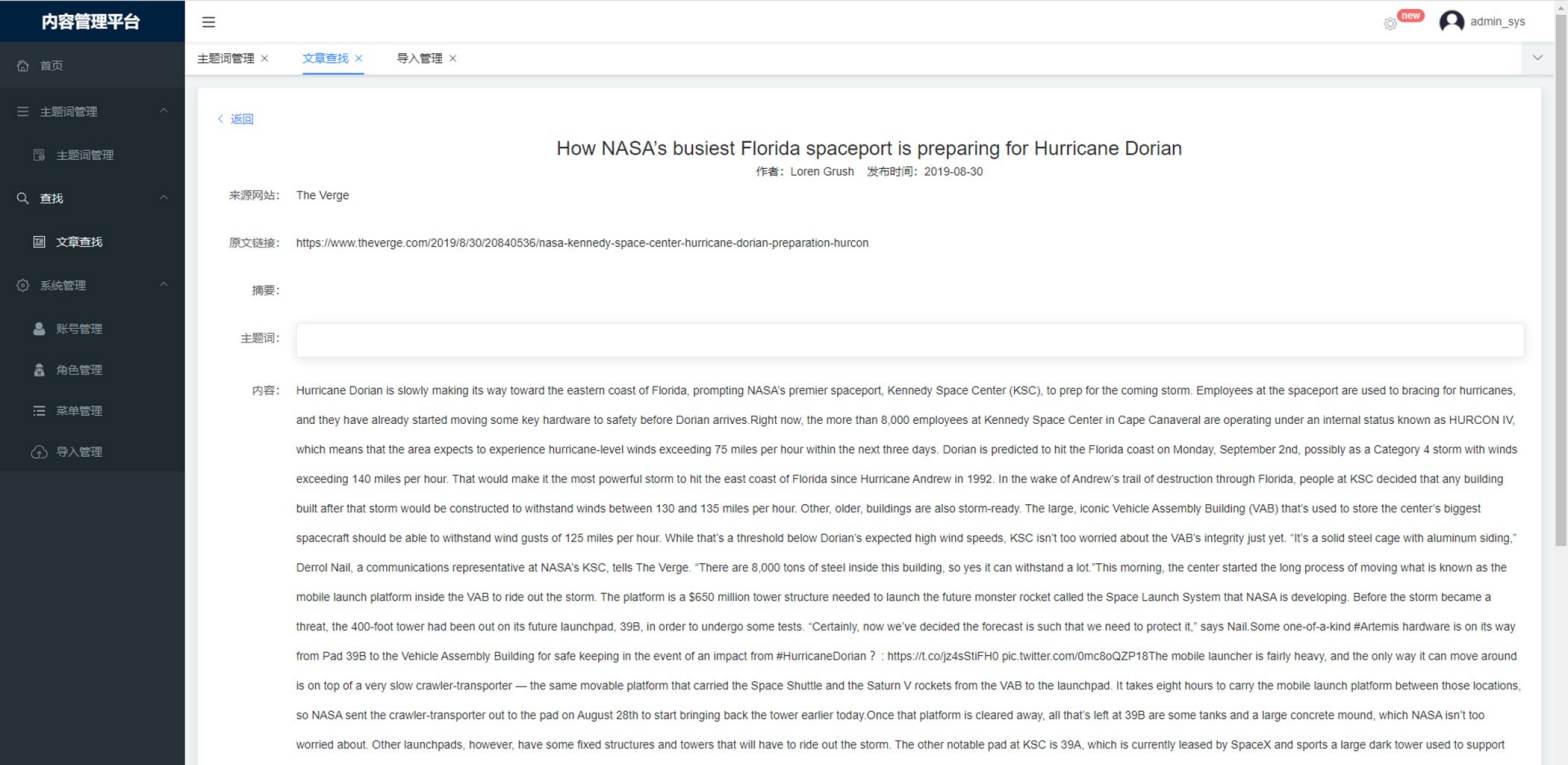
International cutting-edge technology identification article search.

Fifth, data management and visualization layer: This layer manages scientific and technological subject terms, article searches, information import and other services on the "content management platform," as shown in **Figs 10 and 11**.

## 4 Results

After the comprehensive use of a variety of information analysis methods, we now analyze the top 10 international cutting-edge technologies.

### 4.1 6G

In March 2019, the Federal Communications Commission (FCC) opened the 95GHz to 3THz frequency bands for experiments. In July 2019, the Nano-scale Communication Integrated Circuit Laboratory of the University of California, Irvine, USA developed a miniature radio chip with an operating frequency of 115-135Ghz and a data rate of 36 gigabytes per second within 30cm [53].

In March 2019, researchers at the University of Wuppertal in Germany built a complete signal receiving and transmitting system based on silicon germanium (SiGe) materials, which can achieve 260GHz terahertz communication within a distance of 1m [54].

In January and June 2019, South Korean electronics giants LG and Samsung were reported to have established 6G R&D centers [55].

In March 2019, Hiroshima University implemented the first 300GHz terahertz communication in the world based on CMOS low-cost technology [56].

In March 2019, China’s Huawei proposed that 6G should include sea, land, air and even underwater spaces. Tsinghua University also proposed that electric and autonomous vehicles can be used as mobile cloud servers or base stations [57].

In the 6G era, the data rate that users actually experience will reach 10Gbps-11Gbps, which is 100 times the download speed of 5G. It will truly achieve information breakthroughs in time and space, the network will be closer to things, and seamless integration of people and things will be realized [58]. At that time, smart societies, smart cities, smart homes, etc. will be further developed.

### 4.2 Cryptocurrency

As a type of digital currency, cryptocurrency is a transaction medium that uses cryptographic principles to ensure transaction security.

DigiCash, founded in 1989, created an anonymous payment protocol based on cryptography, trying to create the world’s first widely used digital currency [59].

In 2009, Bitcoin, which is a peer-to-peer cash payment system [60], became the world’s first cryptocurrency to use blockchain as the underlying technology. The promotion of cryptocurrency will produce a new method of credit derivation. Smart contracts based on cryptocurrency will impact traditional financial services. The promotion of cryptocurrency will also challenge the sovereign currencies of some countries, which may lead to privacy leaks and illegal activities. produce.

In the digital economy era, the existence of digital currency has become inevitable, so it is imperative to improve the regulatory system [61].

### 4.3 Quantum information

Quantum information is mainly based on the coherent characteristics of quantum mechanics, with the help of unique physical phenomena such as quantum superposition and quantum entanglement, to obtain, transmit and process information in a way that cannot be achieved by classical theory [62].

In 2013, the Japanese Ministry of Education, Culture, Sports, Science and Technology established the Quantum Information and Communication Research Promotion Association and the Quantum Science and Technology Research and Development Agency, and it planned to invest 40 billion yen in research and development in the next ten years [63].

In 2014, the United Kingdom established the National Quantum Technology Plan, investing 270 million pounds to establish a research and development center to carry out academic and applied research [64].

In 2016, the European Union launched the Quantum Declaration flagship program, investing 1 billion euros in research and application promotion in the next ten years, and officially launched the first batch of 20 research projects in November 2018 [65].

In June 2018, the United States introduced the National Quantum Initiative Act [66]. It plans to increase the investment of US$255 million per year in the first phase of 2019–2023, for a total of 1.275 billion. In September of the same year, the White House issued the "Overview of the National Strategy for Quantum Information Science." In the past ten years, the United States has continued to support research in various fields of quantum information through projects such as quantum information science and technology development plans.

In China, the University of Science and Technology of China and Alibaba jointly released a quantum computing cloud platform [67]. Huawei announced a cloud platform consisting of a quantum computing simulator and a programming framework. However, China and the US technology giants have a clear gap in terms of product engineering and application promotion. To reduce the gap, Hefei Benyuan Quantum, the first Chinese quantum computing startup, was established in 2017, injecting new impetus into research and applications.

### 4.4 Gene-editing

Gene editing technology is a breakthrough technology comparable to molecular cloning, PCR and other technologies [68]. In 2015, CRISPR topped the list of Science’s Top Ten Annual Breakthroughs in Science [69]. As a new generation of gene editing tools, CRISPR brings new opportunities for the treatment of serious diseases. According to statistics from CITIC, there are 5438 gene editing patents included in the INNOGRAPHY database. In the field of gene editing, universities and research institutes in the United States and China have strong R&D capabilities in this field.

### 4.5 Drug development aided by AI (AI-assisted new drug development)

We searched in the Web of Science database and the INNOGRAPHY patent platform using "KW = artificial*intelligence*drug*discovery" as the search formula and found that drug development aided by AI is an emerging research area. From the perspective of dissertation agencies, comprehensive large-scale institutions such as Harvard University, the National Institutes of Health, and the Chinese Academy of Sciences are dominant in this field. The backbones of China’s research in this field are Mental Health Center affiliated with the Shanghai Jiao Tong University, School of Medicine, the Institute of Image Communication and Network Engineering of Shanghai Jiao Tong University, the School of Economics and Management of Tongji University, and the Liver Cancer Institute of Fudan University. In 2019, Huizhong Medical released the "Global AI New Drug R&D Research Report." There are currently 149 companies in the world that develop new drugs using AI, among which 14 companies are in China mainly in Beijing, Shanghai, Guangdong, Jiangsu, and Zhejiang regions. In terms of financing, according to network data, six Chinese companies have received financing of 250 million US dollars. Of the cumulative investments into the 149 AI-aided R&D companies, 45% are below 10 million US dollars, and only five companies have an accumulated financing amount of more than 100 million US dollars. The attention of investors is likely to increase in the future.

### 4.6 Graphene with a magic angle

In 2010, researchers at Rutgers University in the United States discovered that introducing a specific twist angle between the crystal orientations of two stacked graphene layers can greatly change the electronic properties of the material [70].

In 2018, MIT doctoral student Cao Yuan published a paper in Nature and pointed out that after the angle of the two layers of graphene was deflected, an unusual superconductivity phenomenon appeared under a certain "magic angle" [71]. Consequently, he was selected as the scientific figure of the year by Nature. This discovery was also named the first of the top ten breakthroughs of Physics World. In 2021, Cao Yuan’s fifth innovation published in Nature found that magic-angle twisted trilayer graphene has not only the same superconducting performance as magic-angle twisted bilayer graphene but also stronger controllability of electronic structure and superconducting performance. A series of discoveries of magic angle graphene are expected to be applied in fields such as energy, electronics, environmental sciences in the future, but due to harsh conditions for superconductivity, more research on the theoretical level is currently being carried out.

### 4.7 Perovskite PV cell

For three consecutive times in 2018–2019, the Massachusetts Institute of Technology (MIT) in collaboration with the Korea Institute of Chemical Technology have set a world record for the efficiency of perovskite solar cells [72]. Their latest study uses a unique selective prerequisite dissolution (SPD) strategy to control perovskite. The structure of mineral crystals and materials can maximize the performance and stability of the equipment.

The Swiss Federal Institute of Technology Lausanne introduced a perfluorobenzene unit to synthesize a hybrid 3D/2D device with the highest reported efficiency [73].

The Institute of Semiconductors of the Chinese Academy of Sciences set a world record twice in 2018 using a post-processing hybrid process.

In March 2019, perovskite solar cells once again became the focus of venture capital. Oxford Photovoltaic Co., Ltd. received a D round of financing with an investment amount of 21 million pounds. Hangzhou Sina Optoelectronics Technology Co., Ltd. received another investment of 50 million US dollar.

From an international perspective, the competition for perovskite solar cells has been extremely fierce in the past three years, and the highest efficiency has been constantly updated. Seven research institutions have reported ultra-efficient perovskite cells (above 22.5%) [74]. This shows very strong research and development capabilities.

### 4.8 Autonomous vehicles

Since the 1970s, many auto companies have researched and developed driverless car technologies. According to the "Global Autonomous Driving Technology Invention Patent Ranking" released by IPR daily and the incoPat Innovation Index R&D Center, among the traditional automakers, GM and Ford are in the leading positions [75]. Worldwide, the number of patent applications from Volkswagen, Nissan, BMW, and Daimler ranks among the top 30 for driving technology inventions.

Since 2017, Google, Uber, Ford, General Motors and other companies have successively deployed products and services such as autonomous taxis, buses and flying vehicles. The Dutch company PAL-V that manufactures the world’s first mass-produced flying car PAL-V Liberty began to accept reservations. In California and other regions of the United States, the autonomous "EasyMile Shuttle" bus system has begun to operate.

Studies have shown that advanced intelligent driving assistance technology can help reduce traffic accidents by 50–80 percent [76]. In addition, unmanned driving will necessitate in-depth cooperation between information technology companies, component suppliers and auto companies. The combined forces of intelligence and interconnection technologies will provide numerous business benefits. According to the 2020 Gartner emerging technology maturity curve, the autonomous driving L4 technology is in the disillusioned bubble period, and the autonomous driving L5 technology entered an expected inflation period in 2019 caused by the technology trigger in 2018. The autopilot technology entered an expected inflation period in 2020 [77].

### 4.9 Negative emissions

Negative emissions [78] means that, in the context of climate change, it is necessary to achieve the targets for net zero emission of greenhouse gases proposed by the Paris Agreement.

At present, the most influential zero-emission projects in the world are the Future Gen project in the United States and the Sleipner project in Norway [79]. The research focuses on power plants to test carbon capture and storage systems.

The field of carbon capture and storage is currently led by China with post-combustion capture demonstration projects [80], such as the Huaneng CSIRO, which is a transformation of Huaneng Beijing Gaobeidian Thermal Power Plant. The designed CO2 recovery rate is more than 85 percent, and the annual recycling capacity is 3000 tons. Although the development of carbon capture and storage technology is relatively mature, at this stage, due to the lack of ideal fixed media, the large-scale application of negative emission technology has encountered a bottleneck. Therefore, the focus of future research on negative emission technology is exploration around the fixed ring.

### 4.10 Chip technology

The transformation of industrial structures requires chip technology, and "Made in China 2025" puts it at the top of the key development areas [81]. At present, the United States and South Korea have absolute dominance in the chip market. Chip technology has extremely high technical barriers, and requires investments in human capital, material and long-term technology accumulation. Although China has MediaTek, HiSilicon Semiconductor and other related chip design companies [82], there is still a big gap between domestic chip manufacturing and that of more developed countries.

## 5 Discussion

With the rapid development of deep learning, this paper proposes a set of methods that automatically integrate neural graph networks and active learning to identify frontier hotspots in international Internet technology media. The main contribution of this paper is to expand the analysis methods other than traditional literary analysis and to build a scientific and technological resource management platform for human-computer interaction. We use machine learning technology to regularly track and mine scientific and technological data to detect international cutting-edge technology hotspots accurately.

Comparing the above research results with the "Global Frontier Science and Technology Hotspots" report released by the Shanghai Institute of Science and Technology Information in 2020 and 2021 shows that both findings are highly consistent. In addition, we discussed the results with well-known experts in the industry who agreed with our research results, further verifying the effectiveness and feasibility of the method proposed in this article. It shows that the method can effectively solve the complex pre-defined classification categories in science and technology. The method has been tested and used in the Shanghai Library (Shanghai Institute of Science and Technology Information). Future research should continue to expand the corpus of scientific and technological information. It should also improve the efficiency of machine learning algorithms and explore a more efficient scientific and technological work mode.

In the past, the P、R、F values of CRF, BERT, Bi-LSTM-CRF, and BERT-Bi-LSTM-CRF models were different for different data sets. However, applying the SpaCy model method enables stable and accurate identification of cutting-edge international technologies and significantly reduces the redundant workload of scientific and technological data analysts. Compared with the previous research methods adopted by other information research institutes, the SpaCy model uses big data thinking to provide analysts with a method to process massive data and obtain high-precision results relatively quickly. At the same time, effective text classification can be accomplished with a lower cost of manual participation. Although the datasets selected in this paper have obtained better experimental results in model training, then these datasets are only some representative data extracted from the enormous amount of Internet technology media information. From a theoretical point of view, the text learned by a graph neural network will be influenced by neighboring nodes, and a slight bias may exist. In practice, we provide new ideas for scientific and technological libraries and information and knowledge services. The active learning method proposed in this paper mainly relies on the training matrix of the training set, which cannot reflect the actual results with infinite accuracy. In the future, it will be learned by expanding the sample with error cases of different topics as supplementary samples, which the authors consider an exciting and worthwhile research problem.
